# Insights into pathophysiology, biomarkers, and therapeutics in tauopathies: Proceedings of the Tau2024 Global Conference

**DOI:** 10.1002/alz.70078

**Published:** 2025-05-29

**Authors:** Bess Frost, James B. Rowe, Rufus O. Akinyemi, Jose F. Abisambra, Nicholas J. Ashton, Matthias Brendel, Luc Buée, David Butler, Maria C. Carrillo, Peter Chung, Claire D. Clelland, Sarah L. DeVos, Kristophe Diaz, Rebecca M. Edelmayer, Fanny M. Elahi, Ratnavalli Ellajosyula, Colin Ewen, Igor Camargo Fontana, Marie‐Christine Galas, Oskar Hansson, Günter Höglinger, Kanta Horie, Agustín Ibanez, Linde Jacobs, Mahmoud B. Maina, Maura Malpetti, Eric McDade, Will McEwan, Laia Montoliu‐Gaya, Catherine J. Mummery, Miranda E. Orr, Jonathan D. Rohrer, Amy Rommel, Carlos Sastre, Tara L. Spires‐Jones, Boon Lead Tee, Tim J. Viney, Jamie M. Walker, Susanne Wegmann, Kristin Wildsmith, Ravi Yadav, Simin Mahinrad, Claire Sexton

**Affiliations:** ^1^ Center for Alzheimer's Disease Research, Department of Molecular Biology, Cell Biology and Biochemistry Brown University Providence Rhode Island USA; ^2^ Department of Clinical Neurosciences, MRC Cognition and Brain Sciences Unit, and Cambridge University Hospitals NHS Foundation Trust, Cambridge Biomedical Campus University of Cambridge Cambridge UK; ^3^ Neuroscience and Ageing Research Unit, Institute for Advanced Medical Research and Training, College of Medicine University of Ibadan Ibadan Oyo State Nigeria; ^4^ McKnight Brain Institute, Brain Injury, Rehabilitation, and Neuroresilience Center, Center for Translational Research in Neurodegenerative Disease, Fixel Institute, Department of Neuroscience University of Florida Gainesville Florida USA; ^5^ Department of Psychiatry and Neurochemistry University of Gothenburg Gothenburg Sweden; ^6^ Banner Alzheimer's Institute and University of Arizona Phoenix Arizona USA; ^7^ Banner Sun Health Research Institute Sun City Arizona USA; ^8^ Department of Nuclear Medicine LMU University Hospital Munich Germany; ^9^ German Center for Neurodegenerative Diseases (DZNE) Munich Germany; ^10^ Munich Cluster for Systems Neurology (SyNergy) Munich Germany; ^11^ German Cancer Consortium (DKTK), partner site Munich, a partnership between DKFZ and Ludwig‐Maximilians‐Universität München (LMU) Heidelberg Germany; ^12^ Inserm, CHU‐Lille, Lille Neuroscience & Cognition University of Lille Lille France; ^13^ Neural Stem Cell Institute Rensselaer New York USA; ^14^ Medical & Scientific Relations Alzheimer's Association Chicago Illinois USA; ^15^ Department of Physics and Astronomy, Department of Chemistry, and Alfred E. Mann Department of Biomedical Engineering University of Southern California Los Angeles California USA; ^16^ Weill Institute for Neurosciences University of California San Francisco San Francisco California USA; ^17^ Memory & Aging Center University of California San Francisco San Francisco California USA; ^18^ Cure.Bio Boston Massachusetts USA; ^19^ CurePSP, Inc New York New York USA; ^20^ Departments of Neurology, Neuroscience, Pathology, Molecular and Cell‐Based Medicine Icahn School of Medicine at Mount Sinai New York New York USA; ^21^ James J. Peters Department of Veterans Affairs Medical Center Bronx New York USA; ^22^ Cognitive Neurology Clinic Manipal Hospital, and Annasawmy Mudaliar Hospital Bengaluru Karnataka India; ^23^ Manipal Academy of Higher Education (MAHE) Manipal Karnataka India; ^24^ UCB Pharma Slough UK; ^25^ Inserm, CHU Lille, CNRS, LilNCog‐Lille Neuroscience and Cognition University of Lille Lille France; ^26^ Lund University Lund Sweden; ^27^ Department of Neurology LMU University Hospital, Ludwig‐Maximilians‐Universität (LMU) München Munich Germany; ^28^ The Tracy Family SILQ Center & Department of Neurology Washington University School of Medicine St Louis Missouri USA; ^29^ Eisai Inc. Nutley New Jersey USA; ^30^ Latin American Brain Health Institute (BrainLat) Universidad Adolfo Ibáñez (UAI) Peñalolén Santiago Chile; ^31^ Global Brain Health Institute (GBHI.org) University California San Francisco (UCSF) San Francisco California USA; ^32^ Global Brain Health Institute (GBHI) Trinity College Dublin Dublin Ireland; ^33^ Cure MAPT FTD Denver Colorado USA; ^34^ Sussex Neuroscience, School of Life Sciences University of Sussex Falmer UK; ^35^ Biomedical Science Research and Training Centre Yobe State University Damaturu Nigeria; ^36^ Department of Clinical Neurosciences and Cambridge University Hospitals NHS Trust University of Cambridge Cambridge UK; ^37^ UK Dementia Research Institute University of Cambridge Cambridge UK; ^38^ Department of Neurology Washington University St. Louis Missouri USA; ^39^ Department of Psychiatry and Neurochemistry, Institute of Neuroscience & Physiology The Sahlgrenska Academy at the University of Gothenburg Gothenburg Sweden; ^40^ Dementia Research Centre, Institute of Neurology University College London London UK; ^41^ Department of Internal Medicine Wake Forest School of Medicine Winston‐Salem North Carolina USA; ^42^ Salisbury VA Medical Center Salisbury North Carolina USA; ^43^ Rainwater Charitable Foundation Fort Worth Texas USA; ^44^ Global Medical Affairs Ferrer Barcelona Spain; ^45^ Centre for Discovery Brain Sciences University of Edinburgh Edinburgh UK; ^46^ UK Dementia Research Institute University of Edinburgh Edinburgh UK; ^47^ Department of Pharmacology University of Oxford Oxford UK; ^48^ Department of Pathology, Molecular and Cell‐Based Medicine Icahn School of Medicine at Mount Sinai New York New York USA; ^49^ German Center for Neurodegenerative Diseases Berlin Germany; ^50^ Einstein Center for Neurosciences Berlin Charité ‐ Universitätsmedizin Berlin Berlin Germany; ^51^ Department of Neurology National Institute of Mental Health and Neurosciences (NIMHANS) Bengaluru Karnataka India

**Keywords:** Alzheimer's disease, neurodegenerative disease, tau disorder, tau protein, tauopathies

## Abstract

Recent years have seen major advances in tau‐associated brain disorders through interdisciplinary research spanning molecular biology, neuroimaging, clinical trials, and therapeutic development. The Tau2024 Global Conference, hosted by the Alzheimer's Association, CurePSP, and Rainwater Charitable Foundation, showcased these efforts by bringing together researchers and experts worldwide to discuss the latest advancements in tau research. The conference aimed to attract talent and funding to study tauopathies, particularly among early‐career researchers, and to foster interdisciplinary alignment and collaboration around challenges in tau research. In this manuscript, we summarize proceedings of the Tau2024 Global Conference, covering a wide range of topics, including lived experiences of individuals with genetic forms of tauopathies, global perspectives on tauopathies, and molecular mechanisms, brain microenvironments, biomarker developments, clinical trials, and therapeutic approaches to tauopathies. Through international, collaborative efforts, innovative research, and a commitment to inclusivity, researchers worldwide have demonstrated transformative breakthroughs toward diagnosing, treating, and, ultimately, preventing tau‐related diseases.

**Highlights:**

- The Tau2024 Global Conference presented updates and advances in tau research.
- Blood‐based biomarkers offer specificity and longitudinal monitoring capabilities.
- There are a range of targetable mechanisms in the cascade of pathogenesis.
- International collaboration is vital to address disparities in tauopathies.

## 1 INTRODUCTION

Recent years have witnessed significant strides in understanding the pathophysiology, biomarkers, diagnosis, and treatment of disorders involving microtubule tau‐associated brain disorders, fueled by interdisciplinary research efforts spanning molecular biology, neuroimaging, clinical trials, and therapeutic development. The Tau2024 Global Conference exemplified these efforts by bringing together researchers and experts worldwide to discuss the latest advancements in tau research. The Tau2024 Global Conference was hosted by the Alzheimer's Association, CurePSP, and Rainwater Charitable Foundation and aimed to attract talent and funding to the study of tauopathies, particularly among early career researchers, and to foster interdisciplinary alignment and collaboration around challenges in tau research.

Abnormal processing, modification, and aggregation of tau protein in neurons and glial cells is central to the pathogenesis of several major neurodegenerative diseases, collectively known as “tauopathies.” Defined by the inclusion or exclusion of exon 10 of the tau microtubule‐binding region, tauopathies are often categorized into three repeats (3R), four repeats (4R), and 3R/4R tauopathies according to the tau isoform present in aggregates. Recent cryo‐electron microscopy (cryo‐EM)‐based analyses provide additional new insights into the ultrastructural folding patterns of fibrillar tau that are specific to distinct tauopathies. Tau pathology is the major neuropathological feature of “primary” tauopathies such as microtubule‐associated protein tau (MAPT)‐related frontotemporal dementia (MAPT‐FTD), progressive supranuclear palsy (PSP), corticobasal syndrome (CBS), and Pick's disease. In “secondary” tauopathies such as Alzheimer's disease (AD), Lewy body dementia (LBD), Parkinson's disease (PD), and Down syndrome,^1^, ^2^ tau pathology develops alongside other pathologic proteins. The study of tauopathies is challenged by the complexity of the tau protein itself, the clinical and molecular heterogeneity of tauopathies, the need for early diagnostic biomarkers, and species differences that limit the translation of preclinical discoveries into clinical practice.

Here, we present the Tau2024 Global Conference proceedings with discoveries and insights across a wide range of topics, including the lived experiences of people affected by tauopathies, global perspectives on tauopathies, pathological and molecular mechanisms of tauopathies, biomarkers, clinical trials, and therapeutic approaches.

## 2 LIVED EXPERIENCE OF INDIVIDUALS WITH GENETIC FORMS OF TAUOPATHIES

The conference opened with a narrative of a family member and caregiver of someone with MAPT‐FTD, who is themselves a carrier of a *MAPT* gene mutation. MAPT‐FTD is a rare form of FTD linked to mutations in the *MAPT* gene and presents with distinct clinical manifestations, including changes in behavior, motor function, memory, and/or language, and is less common than other tauopathies. At the conference it was highlighted that individuals affected by these debilitating diseases face many challenges, depicting a journey spanning generations marked by delayed diagnoses, financial strains, legal battles, and emotional upheaval. Despite these challenges, the families are dedicated to advocacy and research, exemplified in the establishment of Cure MAPT FTD, a non‐profit organization committed to raising awareness and offering support to affected families. The pivotal role of genetic testing and the autosomal dominant nature of the *MAPT* gene mutation was discussed, and concerns about the future roles of their children as potential caregivers were voiced. This narrative demonstrated the pressing need for progress in the diagnosis, prevention, and cure of this devastating neurodegenerative disease.

## 3 GLOBAL PERSPECTIVES ON TAUOPATHIES

Recognizing the worldwide impact of tauopathies, the conference dedicated the first scientific session to the global research landscape, providing an overview of tauopathies across diverse regions, including South America, Africa, India, and the United States. This global perspective underscores the urgent need for tailored research approaches that consider socioeconomic, genetic, and linguistic diversity to develop more personalized and effective interventions for tauopathies, with sensitivity to cultural and economic differences around the world. Key points from these discussions are summarized below.

### 3.1 Tau disorders in Latin America

The prevalence of tau‐related dementia among Latinos in the United States and Latin America ranges from 5.5% to 13.2%,^3^, ^4^ influenced by factors such as age, socioeconomic status, gender disparities, structural disadvantages, and significant underdiagnosis. Unique genetic and environmental interactions,^5^ as well as the presence of large families with typical and atypical mutations,^6^, ^7^ highlight the heterogeneous nature of dementia within Latino populations. Structural barriers, including inadequate healthcare access and cultural stigmas, exacerbate disparities and impose substantial burdens on families. Diagnostic challenges are particularly pronounced in Latin America for frontotemporal lobar degeneration (FTLD), where misdiagnoses often persist for over a decade and are commonly mistaken for psychiatric disorders.

FTLD research has been significantly limited among Latino communities. The Multi‐Partner Consortium to Expand Dementia Research in Latin America (ReDLat) was established to characterize and identify novel inroads for treating FTLD and AD among underserved Latin American populations. Given the socioeconomic,^8^ genetic,^9^ and accelerated brain aging^10^ disparity and diversity inherent in these communities, tailored approaches to understanding dementia are essential. ReDLat was established to bridge this gap by investigating genetic factors and socioeconomic disparities affecting FTLD and AD, with funding from the National Institute on Aging (NIA), Alzheimer's Association, the Rainwater Charitable Foundation, Global Brain Health Institute, National Institutes of Health‐Intramural Center for Alzheimer's and Related Dementias (NIH‐CARD), Alector, Takeda Pharmaceuticals, and the Bluefield Foundation.

The first cohort studied by the consortium included over 4000 participants from 13 centers across seven countries. This effort expanded our knowledge of socioeconomic disparities in clinical and imaging phenotypes, developed robust computational models, and addressed genetic–environmental interactions^10^ with unique datasets from diverse Latino populations. The project's expansion (ReDLat2) will aim to enroll 3000 new participants, focusing on socioeconomic disadvantage and familial presentations to develop tailored models assessing multimodal diversity in Latino populations. Preliminary results from ReDLat2 provide insights into FTLD, emphasizing the impact of genetic and socioeconomic diversity on multimodal phenotypes^11^ and allostatic interoceptive overload,^12^ identifying rare and common variants within large families, and developing computational models of brain clocks for accelerated aging.^10^ ReDLat data have also revealed that the biological embedding of educational disparities is stronger in Latin America than in the United States (US), as observed in more accentuated brain atrophy and reduced functional connectivity, although the effects in FTLD were lower than in AD.^13^ In another study, multiple physical (e.g., pollution) and social (e.g., socioeconomic disparities) exposome factors were linked to accelerated brain age in AD and FTLD, with a stronger impact in Latin America than in other regions.^14^ These efforts underscore the complex interplay of socioeconomic, genetic, and brain diversity in shaping FTLD phenotypes. Overall, the consortium advances scientific understanding of tauopathies and paves the way for better disease and therapeutic models for Latino communities, ensuring a more inclusive approach to the multifaceted nature of dementia.

### 3.2 Speech and language impairments in frontotemporal disorders across languages and geographic regions

The vast linguistic diversity worldwide, spanning over 7000 languages, results in varying clinical manifestations of tauopathies, including individuals living with primary progressive aphasia (PPA). PPA is a heterogeneous group of dementias characterized by a progressive decline in language abilities, affecting individuals’ capacity to speak and communicate effectively. Although nearly 83% of the world's population does not utilize English as their first or second language, most research on speech and language impairments in FTD primarily involves English‐speaking individuals.^15^ This raises uncertainty regarding the generalizability of PPA findings – and diagnostic features ‐ to people using languages with linguistic structures divergent from English.

To address this knowledge gap, examples of language‐specific symptomatology were presented at the conference. For instance, in tonal languages such as Mandarin and Cantonese, people with non‐fluent/agrammatic variant PPA – characterized by difficulty in grammatical comprehension and expression, as well as impairments in speech sound production – showed a higher incidence of tone errors. The ability to produce accurate tone positively correlates with the left anterior insula, a critical area for motor speech programming.^16^ Meanwhile, the nuanced structure of Chinese as a classifier language presented distinct challenges for people with semantic and logopenic variant PPA – marked by difficulty in word comprehension and retrieval – particularly in noun classifier production, a function that positively correlates with anterior temporal lobes.^17^ In Turkish, an agglutinative language known for its high morpheme‐to‐word ratio, challenges in auditory‐verbal short‐term memory emerged due to the complexity and length of words. Unlike English, proficiency in word repetition depended not only on word count and semantic coherence but also on syllable count.^18^ Together, these examples illustrated how linguistic structure can affect clinical presentations in PPA, underscoring the importance of accounting for language diversity in research to improve diagnostic accuracy and culturally relevant care.

These findings underscore the necessity of considering linguistic diversity in research, especially in disorders such as FTD, to prevent misdiagnosis and global care disparities. To address this, the International Network for Cross‐Linguistic Research on Brain Health network was established with the aim of fostering cross‐linguistic collaborations. Through the promotion of cross‐linguistic work, cultivation of international collaborations, and co‐establishment of interlinguistic databases, efforts are directed toward enhancing the understanding and enriching the cognitive research landscape with respect to linguistic diversity.^15^

### 3.3 Developing the African Dementia Consortium

While AD is the most common form of dementia in Africa, similarly to other world regions, other tauopathies are less commonly reported. The first case of FTD in western and central Africa was documented in 2006, while a review of hospital records showed that FTD accounted for 3.7% and 1.8% of dementia cases at two memory clinics in Nigeria and South Africa, respectively.^19^, ^20^ Neuroimaging‐confirmed cases of PSP are rare, with only a few anecdotal reports available.^21^ In addition, nodding syndrome – a unique neurological disorder prevalent in parts of Eastern Africa – primarily affects children and is characterized by repetitive head nodding, potentially progressing to grand mal seizures.^22^ Previously thought to be an autoimmune neuroinflammatory disorder linked to *Onchocerca volvulus* infection,^23^ evidence from recent neuropathological studies suggests that it is a form of tauopathy.^24^, ^25^ Specifically, neuropathologic findings in individuals with fatal nodding syndrome have identified tau‐positive neurofibrillary tangles (NFTs), pre‐tangles, and neuropil threads primarily in the frontal and temporal lobes, as well as in brainstem regions such as the substantia nigra and locus coeruleus, indicating that tau pathology underpins this unique disorder.

The need to enhance global diversity, equity, and inclusion in dementia research, particularly by involving underrepresented African populations in dementia genetics and genomics, led to the establishment of the African Dementia Consortium (AfDC).^26^, ^27^ AfDC is an Africa‐led international research consortium that brings together African dementia researchers in a multidisciplinary framework to generate clinical, cognitive, socioeconomic, neuroimaging, and multi‐omics datasets to improve the characterization of dementia phenotypes among Africans; identify protective and risk factors and unmask the cognitive trajectories of different dementia subtypes. AfDC also aims to contribute knowledge on diagnostic and prognostic biomarkers and enhance the development of personalized interventions for prevention and treatment. Research findings from AfDC are expected to enhance the translation of evidence to policy and practice for the promotion of brain health, dementia risk reduction, and developing national and regional dementia plans. Together, these efforts are focused on mitigating the rising burden of dementia among Africans and those of African ancestry in the diaspora, ultimately contributing to the reduction of global dementia.^28^

### 3.4 Clinical phenotypes and genetics of PSP: an Indian perspective

Based on the global prevalence estimates of PSP, India (home to 1.4 billion people) has a significant burden of PSP (betwen14,400 and 259,000 persons).^29^ There are no epidemiological studies available, but a large hospital‐based study from three cities across India found Parkinsonism in 9460 (64.9%) people among 14,561 persons diagnosed with movement disorders.^30^ PD (65.8%), followed by PSP and CBS (11.6%) and multisystem atrophy (6.6%), were the common phenotypes, while a substantial number (15.9%) were either atypical or other forms of tauopathy. A recent retrospective analysis of 334 people with PSP in India has provided valuable insights into the phenotypic presentation of the disease, showing that the most common subtype was PSP‐Richardson syndrome (PSP‐RS), accounting for 72% of cases.^31^ Consistent with studies from Europe and North America, people with PSP‐Parkinsonism in India tend to reach milestones such as wheelchair dependency, unintelligible speech, and dysphagia later than those with other subtypes.^31^

Genetic studies on PSP in India are limited, with research exploring various genetic markers associated with the condition such as C9orf72^32^ and *MAPT* mutations. Aswathy et al. evaluated *MAPT* genetic variations in a cohort of South Indian people with FTLD, finding no pathogenic mutations but identifying several non‐pathogenic SNPs, including a novel intronic variation, IVS9‐48.^33^ Additionally, Dey et al. analyzed 106 people with PSP for pathogenic *MAPT* gene variants and SNPs within the *MAPT*, *STX6*, *MOBP*, and *EIF2AK3* genes. They found significant associations with several SNPs and *MAPT* sub‐haplotypes, as well as connections between certain genotypes and clinical outcomes such as age of onset, cognitive function, and disease severity.^34^ Together, these findings highlight the need for larger, locally representative cohorts with longitudinal follow‐up to better understand PSP's phenotypic and genetic variations in India.

## 4 BRAIN MICROENVIRONMENT IN TAUOPATHIES

The brain microenvironment comprises various elements, including neurons, glial cells, and vascular structures that engage in complex cellular and molecular interactions. This dynamic milieu is crucial in maintaining brain function and hemostasis, influencing processes such as neuronal signaling and immune responses. Disruption of this finely tuned microenvironment can contribute to various neurological disorders, including tauopathies.^35^ To understand the dynamics of this microenvironment in the context of tauopathies, the conference dedicated a session to brain microenvironments. Discussions ranged from spatial proteomic comparisons of tauopathies, microvascular contributions to pathological progression of tauopathies, mechanisms of trans‐synaptic propagation of tau pathology, and the vulnerability profile of glutamatergic presynaptic terminals. These discussions aimed to highlight the interplay of various components within the brain microenvironment and their alterations under pathological conditions associated with tauopathies. Key points from these discussions are summarized in what follows.

### 4.1 Spatial proteomic comparison of tauopathies

The development and distribution of phosphorylated tau (p‐tau) in the hippocampus and entorhinal region in AD differs from that of primary age‐related tauopathy (PART)^36^ and is directly related to the burden of amyloid beta (Aβ) present in the hippocampus.^37^ To understand the mechanisms underlying the differences in p‐tau distribution and the resistance to Aβ plaque deposition, researchers at the conference discussed NanoString GeoMx digital spatial profiling (DSP) that was utilized to perform spatial proteomics on hippocampi of people with AD and PART pathology. Regions of interest (ROIs) for spatial proteomic analysis included NFTs, normal neurons, and their microenvironments in the hippocampal cornu Ammonis (CA) subregions and the entorhinal cortex. Analyses revealed higher levels of proteins that may reduce or prevent Aβ plaque deposition in individuals with PART, such as Insulin‐Degrading Enzyme, neprilysin, and Cathespin D. Individuals with PART also displayed higher levels of synaptic and dendritic markers in the CA1 subregion and entorhinal cortex but not in CA2, which houses the highest burden of NFTs in PART. In addition, many proteins involved in proteostasis were higher in those with lower Thal phase (Thal phases describe the progression of Aβ plaque deposition in the brain, with lower phases indicating limited deposition and higher phases representing more widespread distribution), suggesting better degradation of misfolded or aggregated proteins. Interestingly, it was also apparent that individuals with possible PART (those with Thal phases 1 and 2) displayed protein expression patterns that more closely resemble AD rather than definite PART (Thal phase 0), supporting the notion that “possible PART” may represent early AD, whereas “definite PART” could be a distinct entity.^38^

### 4.2 Microvascular pathological contributions to tauopathies

The accumulation of tau in neurons and/or glia is a hallmark of primary tauopathies.^39^ The process by which the brain transitions from normal functioning to a state of dysfunction, characterized by the misfolding and aggregation of proteins such as tau, remains poorly understood. In addition, cerebral small vessel diseases (cSVDs) may contribute to the onset and progression of tauopathy across different disease subtypes.^40^, ^41^, ^42^, ^43^ Blood vessels remain an accessible target for therapeutic intervention and hold significant promise for therapeutic development in tauopathies.

cSVDs impact the neuro‐glial microenvironment and function, referred to collectively as vascular contributions to cognitive impairment and dementia (VCID).^43^ At the conference, researchers discussed molecular pathologies in cSVDs that contribute to the development and progression of neuro‐glial tauopathies, with a specific focus on brain arteriolosclerosis (BASC) contributions to dementia. BASC is strongly associated with hypertension and cerebral autosomal dominant arteriopathy with subcortical infarcts and leukoencephalopathy (CADASIL), a monogenic form of VCID caused by mutations in the *NOTCH3* gene. Proteomic analysis of biofluids from people with CADASIL revealed dysregulation of vascular plasticity, fibrosis, and immune function.^44^, ^45^ Preliminary studies suggested that multimodal models are associated with enlarged perivascular space (ePVS), measures of white matter injury, and molecular measures of immune dysfunction and fibrosis. Furthermore, leveraging single nucleus RNA sequencing datasets, blood proteomic signatures map to specific cell types such as vascular smooth muscle cells, oligodendrocytes, and T cells. Overall, this work is expected to generate high‐resolution molecular data from human biospecimens and cell models for the data‐driven discovery of BASC‐VCID molecular contributions to tauopathy.

### 4.3 Trans‐synaptic propagation mechanisms of pathological tau

Tau pathology accumulates in the brain in a stereotypical spatiotemporal pattern as the disease progresses in AD and PSP.^46^, ^47^ One potential mechanism of the spread of tau pathology is the trans‐synaptic propagation of pathological forms of tau. In animal models of tauopathy, tau pathology can spread between brain regions via synaptic connections.^48^, ^49^, ^50^, ^51^, ^52^, ^53^ Injection of extracts of AD or PSP brain into mice expressing human tau also induces the formation of tau aggregates, which then spread through the brain.^54^ The combination of tau positron emission tomography (PET) and functional connectivity imaging in humans indicates that tau pathology accumulates in functionally connected brain circuits.^55^, ^56^, ^57^

While imaging tau within individual synapses in the human brain remains challenging, resulting in a lack of direct evidence regarding tau accumulation inside synapses and its trans‐synaptic spread in humans, high‐resolution array tomography and immuno‐electron microscopy demonstrate that oligomeric tau accumulates in pre‐ and postsynaptic terminals in AD.^58^ Further, presynaptic oligomeric tau has been observed in brain areas with limited NFT pathology, indicating synaptic oligomeric tau precedes tangle formation. Moreover, oligomeric tau has been observed in both pre‐ and postsynaptic terminals in PSP.^46^ In addition, a novel living human brain slice model has been developed utilizing organotypic brain slices derived from peritumor cortical tissue resected during glioblastoma debulking surgery. These human brain slice cultures (HBSCs) were challenged with tau isolated from human *post mortem* PSP brain tissue. Treatment of live human brain slices with tau‐containing PSP brain extract led to postsynaptic uptake of tau oligomers and astrocyte engulfment of synapses.^59^ Together, these data suggest that tau pathology may spread through the brain by oligomeric tau transferring across synaptic connections.

### 4.4 Vulnerability of subcortical presynaptic terminals to tau pathology

Spatial navigation and orientation rely on a combination of egocentric (body‐derived) and allocentric (landmark‐based) cues. This ability can be disrupted, leading to disorientation, which is recognized as an early sign of dementia.^60^ In 1991, H. Braak and E. Braak demonstrated that the anterodorsal thalamus (ADn) – a key part of the Papez circuit responsible for spatial navigation and orientation – is highly vulnerable to early tau pathology and neurodegeneration.^47^, ^61^ In line with this, research presented at the conference showed that misfolded pathological forms of tau (p‐tau) are detectable in the ADn of human *post mortem* tissue not only in Braak stages I to VI but also in “pre‐Braak” stage 0.^62^ The ADn consistently exhibited p‐tau pathology, while adjacent thalamic nuclei were affected only in more advanced Braak stages. Further, preliminary findings using electron microscopic identification of p‐tau suggested a preferential spread of p‐tau between vesicular glutamate transporter type 2‐containing subcortical terminals and ADn dendrites rather than spreading anterogradely via cortical terminals.^62^ Given the ADn's dense population of head direction cells,^63^ its heightened vulnerability to p‐tau likely contributes to disorientation. Leveraging this knowledge could aid in identifying individuals at risk of developing memory impairments, facilitating earlier intervention strategies.

## 5 MOLECULAR MECHANISMS DRIVING TAU‐INDUCED NEURODEGENERATION

Understanding the intricate molecular and pathological mechanisms underlying tauopathies is crucial for early detection and developing effective therapeutic strategies, ultimately offering hope for improved outcomes and quality of life for individuals affected by these disorders. The conference provided a comprehensive exploration of molecular mechanisms of tauopathies, focusing on aspects that are readily translatable to clinics. Key topics discussed included implications of guanine quadruplex‐structured DNA in tau pathology, maladaptive translation processes in tauopathies, leveraging genetic code expansion for expression and purification of hyperphosphorylated tau, and intracellular degradation pathways of tau, as summarized below.

### 5.1 Role of neuronal guanine quadruplex‐structured DNA in tau pathology

In addition to its well‐known double helical structure, DNA exhibits alternative secondary configurations such as guanine quadruplex (G4) structures. G4 structures play crucial roles as regulators of gene expression and genomic stability and influence protein homeostasis.^64^ Despite their significance, the association of G4 structures with neurodegenerative diseases has been largely overlooked. Recent research has shed light on this relationship, identifying the presence of DNA fragments within sarkosyl‐insoluble aggregates, including tau protein, in the brains of individuals with AD. The DNA contained within these aggregates is enriched in sequences predicted to adopt G4 structures, suggesting that G4‐structured DNA (G4 DNA) may contribute to the pathology of AD.^65^

Tau pathology strongly impacts the dynamics of nuclear G4 DNA. Early stages of the disease are characterized by impaired nuclear distribution of G4 DNA, with accumulation of G4 DNA in the cytoplasm of neurons exhibiting hyperphosphorylated or oligomerized tau and oxidative DNA damage. Research has shown that the altered distribution of G4 DNA persists in later stages of pathology, even in the presence of larger tau aggregates. In addition, it was demonstrated that tau pathology‐induced G4 DNA redistribution was associated with changes in the size of nuclei and nucleoli, indicative of stress responses and the activation of pro‐survival mechanisms.^64^ Together, these findings provide insight into the involvement of G4 DNA structures and nuclear and nucleolar mechanobiology in the etiopathogenesis of tauopathies,^66^ paving the way for further exploration of their roles in disease development and progression.

### 5.2 Maladaptive translation processes in tauopathies

Numerous tau‐interacting proteins are involved in RNA translation,^67^ yet the role of tau in this process remains elusive. Researchers have investigated the association of tau with RNA, ribosomes, and nascent proteins across various models of tauopathies ranging from cell‐free systems to examinations of human brains.^68^ Interestingly, tau was found to drive ribosomal selectivity, leading to an *adaptive translatome*. In disease states, however, tau disrupts this adaption, leading to an aberrant translatome incapable of mitigating neuronal dysfunction.^69^ To better understand the role of tau‐associated RNAs, crosslinking immuno‐precipitation (eCLIP) using total and disease‐associated p‐tau antibodies can be employed. Preliminary data presented at the conference suggested that the association of tau with coding RNAs increases as AD progresses, and several patterns of translational regulation unique to different transcripts were identified. Moreover, the potential of rebalancing the translatome to restore brain function was explored. Building upon prior evidence that pathological tau activates PERK (a kinase promoting translational repression^70^), a PERK inhibitor was administered to tau transgenic mice, which protected the brains from the deleterious effects of tauopathies.^71^ Together, these findings suggest that physiological forms of tau play an important role in regulating adaptive responses to cell stress and that they are less effective at modulating translation to adapt to cellular stress.

### 5.3 Molecular interplay between phosphorylation and tau fibrillization with genetic code expansion

While the etiology of AD is linked to the presence of NFTs (composed of paired helical filaments [PHFs]), the inability to recreate tangles in the laboratory has hindered efforts to understand their formation or to investigate therapeutic compounds that target them. While PHFs are composed of hyperphosphorylated full‐length tau, attempts to reconstitute AD‐like PHFs from full‐length tau in cell‐free conditions have been challenging.^72^ It has been proposed that the conformational flexibility of the domains flanking the aggregation‐prone region of tau prevents PHF formation. If these domains were to be phosphorylated, the resultant loss in conformational flexibility could lead to increased PHF formation. By using genetic code expansion to express proteins harboring the non‐canonical amino acid phosphoserine,^73^ preliminary results presented at the conference suggested that p‐tau was successfully purified, demonstrating increased tau aggregation, with aggregates possessing structural properties akin to PHFs. These data suggest that abnormal phosphorylation of tau may precede and be necessary for the development of PHFs and the subsequent formation of NFTs. Although integrating multiple phosphoserines remains an experimental bottleneck, this strategy may enable the expression and purification of hyperphosphorylated, full‐length tau.

### 5.4 Intracellular degradation of tau assemblies via cytosolic antibody receptor TRIM21

The assembly of tau into multimeric species results in a toxic gain of function and compromises the functionality of the native protein. The E3 ligase known as TRIM21 is of particular relevance to tau aggregation,^74^ as it becomes selectively activated upon clustering,^75^ enabling selective targeting and degradation of assembled tau variants. A recent study demonstrated that the fusion of nanobodies that bind to tau^76^ and to the catalytic RING domain of TRIM21 promoted the efficient degradation of filamentous aggregates in cell‐based and neuronal models.^77^, ^78^ Soluble monomeric tau was not degraded, confirming the aggregate selectivity of the approach. Administering these constructs to animal models of tau pathology resulted in a reduction of insoluble, hyperphosphorylated, and seed‐competent tau species.^77^, ^78^ Therefore, this work suggests that tau aggregates can be selectively removed from the brain while sparing functional monomers. The therapeutic potential of these constructs by inducing tau degradation is under investigation.

## 6 DISCOVERY AND DEVELOPMENT OF BIOMARKERS

Biomarkers are critical tools for diagnosis, stratification by prognosis or phenotype, tracking disease progression, and evaluating therapeutic efficacy in clinical trials and real‐world settings. In the context of tauopathies, identifying precise and reliable biomarkers is essential. The conference dedicated a session to recent advancements in biomarker research, focusing on topics such as biomarkers of inflammation, tau PET imaging, and fluid biomarkers, as summarized below.

### 6.1 Novel tau‐PET biomarkers

Tauvid (formerly also known as flortaucipir, AV1451, T807) is the only FDA‐approved tau tracer. Its clinical use has been limited to the diagnosis of AD, and research applications have been affected by low affinity for non‐AD tau and off‐target binding. Second‐generation tau tracers such as MK‐6240 and PI‐2620 have been developed with improved selectivity and binding to AD‐associated brain patterns.^79^ However, with the possible exception of PI‐2620 and PM‐PBB3, it has proven difficult for PET ligands to reliably and sensitively detect 3R or 4R tau isoforms in the context of non‐AD primary tauopathies (FTD, PSP, CBD).^80^, ^81^

While tau‐PET with [^18^F]PI‐2620 has emerged as a valuable biomarker in distinguishing the 4R tauopathies of PSP and CBS from healthy and disease controls,^80^, ^82^, ^83^, ^84^ the translation of in vitro 4R‐tau binding^85^ to in vivo tau‐PET signals remains unclear. Research presented at the conference indicated that using optimized reference tissues in the temporal lobe and the cerebellar crus enhances the correlation between [^18^F]PI‐2620 signals and clinical severity of PSP, potentially attributed to tau pathology accumulation in white matter branches of the cerebellum during later disease stages.^46^ Furthermore, in clinical settings, it has been reported that dynamic [^18^F]PI‐2620 imaging may facilitate PET‐based A/T/N assessment during a single 1‐h PET session by employing perfusion and efflux indices alongside late‐phase tau binding.^86^, ^87^, ^88^

A longitudinal [^18^F]PI‐2620 PET/MRI study in mouse models of tauopathy revealed elevated PET signals in the presence of high neuronal tau but low astroglial tau.^89^ Additionally, a novel approach involving cell sorting following radiotracer injection^90^, ^91^ elucidated higher tracer uptake in single neurons compared to astrocytes in PS19 mice (a 4R‐tau mouse model), in line with predominant neuronal tau spread along functionally connected brain regions.^55^ However, autoradiography data of [^18^F]PI‐2620 displayed heterogeneous results with either present or absent tracer binding observed in PSP and CBD target regions.^80^, ^92^, ^93^, ^94^, ^95^ In an autopsy sample, in vivo regional [^18^F]PI‐2620 tau‐PET signals correlated strongly with an abundance of fibrillar tau in a small sample of people with and without PSP.^89^ In another autopsy sample of people with PSP, AT8 tau‐positive neurons, but not astrocytes, were the driver of [^18^F]PI‐2620 autoradiography signals.^89^ These data highlight that neuronal tau constitutes the dominant signal source of [^18^F]PI‐2620 tau‐PET signal increases in 4R‐tauopathies, yielding the capacity to translate to an in vivo signal.

Another tau‐PET biomarker presented at the conference was [^18^F]APN‐1607 (or [[^18^F] florzolotau]) developed by APRINOIA Therapeutics. [^18^F]APN‐1607 is being developed as a novel PET tracer for the detection of mixed 3R/4R, 3R, and 4R tauopathies, including AD, PSP, CBD, and FTD, along with their respective variants, and chronic traumatic encephalopathy.^81^, ^96^ The advent of a selective 4R tau PET tracer would not only serve as a valuable diagnostic tool for the early diagnosis of PSP and other non‐AD tauopathies but also enable the enrichment of therapeutic trials with individuals in the early stages of the disease and the monitoring of treatment effects. Clinical studies, undertaken in vivo but with pathology or genetic validation, are required.

Recent ultrastructural analyses by cryo‐EM have identified specific binding sites of various tau PET probes, including MK6240, GTP1, and florzolotau. Unlike other tracers, the ability of florzolotau to bind multiple beta sheet stacks within groves of tau filaments allows it to detect tau assemblies in non‐AD tauopathies, which have a disrupted J‐shaped cavity. Preclinical studies of florzolotau have shown selective and single‐digit nanomolar binding to 3R and 4R tau fibrils without significant off‐target binding to other brain proteins, including monoamine oxidase A (MAO‐A) and monoamine oxidase B (MAO‐B). Limited cross‐reactivity to amyloid fibrils has been reported in vitro. In addition, florzolotau exhibits binding to tau aggregates in brain tissue from people with AD, PSP, CBD, and Pick's disease, as confirmed by autoradiography and immunohistochemistry.^81^

Several clinical studies have shown that florzolotau can bind to 3R, 4R, or mixed 3R and 4R tau isoforms in diverse tauopathies, including AD, PSP, and CBD, as well as FTD due to *MAPT* mutations.^81^, ^96^ These studies have also shown correlations between standardized uptake value ratio and clinical severity as measured by the CDR sum of boxes in people with AD^81^ and the PSP rating scale in those with PSP.^81^, ^96^

Together, these results suggest that florzolotau may provide a useful PET tracer for the detection of tau assemblies of different isoform types and enable a more accurate diagnosis at earlier disease stages, particularly in PSP.

### 6.2 Fluid biomarkers

#### 6.2.1 CSF biomarkers

Different p‐tau and non‐p‐tau species have been identified in CSF across the AD continuum, showing that distinct tau phosphoepitopes exhibit varying temporal profiles,^97^ and even fragments containing the same phosphorylation could have different emergencies.^98^ Furthermore, recent discoveries highlight multiple microtubule‐binding region (MTBR)‐tau species in human CSF, derived from studies such as the Dominantly Inherited Alzheimer Network, Knight Alzheimer Disease Research Center, and Swedish BioFINDER‐2 study.^99^, ^100^, ^101^

The tau protein comprises multiple repeat domains (R1 to R4) within its MTBR that are associated with its molecular structure and aggregation properties. Research has shown that CSF MTBR‐tau profiles may be specific to each tauopathy and vary by disease stage. For example, CSF MTBR‐tau354 located in the R4 domain is elevated in the CSF of people with AD compared to controls but reach saturation or may even decrease after the onset of symptomatic disease.^101^, ^102^ Conversely, CSF levels of MTBR‐tau243 located in the R1 domain continuously increase through AD progression and show a strong correlation with tau‐PET imaging.^100^, ^102^ In people with non‐AD tauopathy such as CBD, levels of MTBR‐tau275 and 282, which are specific to 4R‐tau isoforms, have been shown to increase in brain samples but decrease in CSF samples compared to control and other primary tauopathies like Pick's disease.^99^ Thus, CSF levels of MTBR‐tau reflect the enrichment profiles in brain tau aggregates, suggesting their potential utility as biomarkers for AD staging and tracking the effects of tau‐targeting therapies. For primary tauopathies, CSF MTBR‐tau 275 and 282 may represent the affirmative biomarkers to aid in diagnosis and facilitate clinical trial designs.

Moreover, recently a novel AD staging system based on CSF biomarkers was proposed incorporating various tau variants and Aβ levels. Machine learning approaches to separate stage and subtypes within a heterogeneous cross‐sectional cohort (SuStaIn) can identify biomarker trajectories across the AD continuum, facilitating the development of a robust staging system necessitating only five biomarkers.^103^ This CSF‐based staging model offers several advantages, including participant stratification based on Aβ and tau pathology as well as neurodegeneration levels without necessitating PET scans, potentially streamlining diagnosis and management processes. Additionally, the model aids in the selection of participants for clinical trials targeting Aβ or tau pathology by predicting longitudinal trajectories of imaging biomarkers based on the baseline CSF stage. Finally, participants classified with higher CSF stages exhibit an increased risk of clinical progression, underscoring the prognostic value of this staging system.^103^

Despite its efficacy, CSF sampling remains relatively invasive and costly, limiting its scalability for large‐scale use. Furthermore, the availability of analysis tools can vary substantially across different settings, impacting access to CSF‐based AD staging systems. To improve clinical accessibility, CSF‐based AD staging systems may be translatable into blood biomarkers. Although some key biomarkers, such as MTBR‐tau243, are still under development for plasma use,^100^ other existing plasma biomarkers may offer viable options that could be more widely accessible on a global scale.^104^

#### 6.2.2 Blood‐based biomarkers

Rapid advances in AD research and diagnosis have led to the identification of specific plasma biomarkers, marking significant progress from earlier global proteomic approaches to targeted and ultra‐sensitive methodologies.^105^, ^106^ In under one decade, these breakthroughs led to the development of multiple immunoassays to quantify tau phosphorylation at specific positions, such as p‐tau181, 217, and 231.^107^ Among these biomarkers, p‐tau217 has emerged as a focal point due to its enhanced disease specificity for AD and its superior sensitivity to dynamic longitudinal change. Moreover, recent advancements have enabled the integration of p‐tau217 detection into fully automated platforms, streamlining the diagnostic process.^108^, ^109^, ^110^

Challenges remain despite significant advancements in plasma tau biomarker development. Physiological factors such as chronic kidney disease, variability in fasting status, and non‐specific binding in certain immunoassay setups can complicate the interpretation of plasma p‐tau217 levels.^111^ Additionally, the ongoing exploration of how to effectively distinguish AD‐specific from non‐AD‐specific pathologies continues to drive research efforts forward. Recent breakthroughs, such as the detection of p‐tau217 from dried blood spots^112^ from the capillary collection, offer promising solutions.^113^ This innovation offers promising early results, opening avenues for global, remote, and regular evaluation of plasma p‐tau217 levels, revolutionizing the accessibility and frequency of AD monitoring.

Immunoprecipitation mass spectrometry (IP‐MS) has been introduced to simultaneously measure different plasma p‐tau and non‐p‐tau species in a single analysis, thereby avoiding platform heterogeneity issues. Initial findings from this method highlighted specific site‐specific phosphorylations – p‐tau231, p‐tau217, and p‐tau205 – as having the most pronounced fold changes along the AD continuum and strongest correlations with tau PET imaging.^114^ The advantage of IP‐MS methods is the ability to calculate the percent p‐tau from the ratio of p‐tau/non‐t‐tau, which seems to be less affected by the challenges mentioned in immunoassays.^115^ Interestingly, these phosphorylated variants displayed differential associations with amyloid and tau PET scans: p‐tau231 exhibited stronger correlations with amyloid, p‐tau217 demonstrated associations with both amyloid and tau, and p‐tau205 primarily correlated with tau levels.^114^ These observations were validated in a neuropathologically confirmed AD cohort,^116^ where p‐tau231, p‐tau217, and p‐tau205 showed the most significant alterations. Notably, plasma p‐tau231 demonstrated the most significant changes with mild Aβ plaque density, while plasma p‐tau217 increased with moderate Aβ plaque density and continued elevation thereafter. Plasma p‐tau205 levels exhibited the most pronounced changes with severe Aβ plaque scores and in advanced Braak stages.^116^ These findings suggest the potential of utilizing plasma tau variants for AD staging, with ongoing efforts being concentrated on validating this approach.

Taken together, the field of AD biomarker research has made significant strides, with a shift toward specific plasma biomarkers such as p‐tau217. The integration of ultra‐sensitive detection methods and automation has enhanced diagnostic accuracy and longitudinal monitoring capabilities. Moving forward, methods such as IP‐MS hold promise in addressing emerging challenges in the field, including determining the impact of comorbidities and copathologies on plasma tau species and understanding the pathophysiology of tau hyperphosphorylation. Moreover, the emergence of dried blood spots and fingerstick collection methods could offer a more accessible and comprehensive approach to AD biomarker assessment, signifying a new era in AD diagnostics.

### 6.3 Biomarkers of inflammation

Preclinical, genetic, and clinical studies indicate brain inflammation as an important pathogenic mechanism in AD, primary tauopathies such as FTD, and related disorders.^117^, ^118^ Recent findings from in vivo PET imaging with tracers binding to TSPO, a marker overexpressed in activated microglia, have demonstrated higher regional inflammation in each of the major tauopathies. The degree of inflammation correlates with clinical severity, and it predicts faster clinical decline in AD,^119^ FTD,^120^ and PSP.^121^ PET measures of inflammation also correlate with *post mortem* immunohistochemistry measures of microglial activation in the same individuals.^122^, ^123^, ^124^ These findings support the role of central nervous system inflammation in accelerating disease progression across neurodegenerative diseases. Building on this, new international collaborations are aiming to develop a pipeline to standardize and apply different PET tracers targeting microglial activation in clinically similar cohorts.

Although PET imaging captures the distribution and quantity of brain inflammation, fluid markers offer greater scalability and repeatability in large populations and clinical trials. There are diverse methods to measure inflammation in peripheral biofluids, including blood, which are clinically relevant and mechanistically informative blood‐based markers in tauopathies. The blood‐based inflammatory profiles reflect both the involvement of immune cells and their chemical signaling patterns (cytokines). For example, in PSP, CBD, FTD, and AD/MCI, a similar transdiagnostic pro‐inflammatory cytokine profile is observed that differentiates affected individuals from controls. Notably, higher pro‐inflammatory profile scores were associated with shorter survival and higher microglial activation in frontal and brainstem regions as measured by TSPO PET.^125^ Characterization of such inflammatory profiles in individuals with neurodegenerative disorders holds the promise of facilitating accessible and scalable biomarker development, enabling personalized medicine approaches, and enhancing clinical trial efficacy.

## 7 CLINICAL TRIAL DESIGN AND ENROLLMENT IN STUDIES OF TAUOPATHIES

Successfully addressing tauopathies requires the capability to confirm the biological disease diagnosis, drawing lessons from anti‐amyloid trials.^126^ Although specific biomarkers for primary tauopathies remain lacking, recent advances offer promise for differentiating between different tauopathies^99^ and tracking tau tangle accumulation in the brain.^100^ Notably, early‐phase trials targeting tau in AD have demonstrated measurable biological changes. For example, BIIB080, an antisense oligonucleotide targeting the *MAPT* transcript gene, has shown a dose‐dependent sustained reduction in tau levels in CSF,^127^ which is associated with reduced tau burden in the brain.^128^ Furthermore, while the dominant approach in tau‐targeted therapy currently involves immunotherapy, the precise tau epitope to target remains under investigation. Recent evidence suggests the potential significance of targeting seed‐competent tau. Promising interim results from tau immunotherapy (E2814) targeting two sites of the MTBR^129^ highlights the potential for second‐generation anti‐tau monoclonal antibodies, building on the growing understanding of tau pathophysiology.

A possible strategy for tackling tauopathies, particularly in AD, involves the joint targeting of both Aβ plaque and tau tangle pathology. The temporal links between the onset of Aβ plaques and the initial increase in p‐tau suggest important mechanistic links between these two proteinopathies in AD.^97^, ^98^ While evidence suggests that neuritic Aβ plaques can trigger tauopathy in AD, recent trials of effective amyloid‐lowering therapies have not resulted in a robust decrease of tau PET signals, which is principally a marker of neurofibrillary tangle burden.^130^, ^131^, ^132^ This implies that a dual approach targeting both Aβ plaque and tau tangles may be more effective in AD but requires robust clinical trial evidence. The recent approvals of anti‐amyloid immunotherapies^131^, ^132^, ^133^ present both pragmatic and mechanistic grounds for considering such dual (or concurrent) amyloid/tau clinical trials. Leveraging the natural history and clinical trial data of gantenerumab (anti‐aggregated amyloid immunotherapy, ClinicalTrials.gov number NCT04623242) and solanezumab (anti‐monomeric amyloid immunotherapy, ClinicalTrials.gov number NCT04623242), the Dominantly Inherited Alzheimer's Network Trials Unit (DIAN‐TU) platform has recently developed a dual‐therapy trial targeting both amyloid and tau (ClinicalTrials.gov number NCT05269394). This trial utilizes both soluble (CSF and plasma) and aggregated biomarkers (tau PET), enabling researchers to account for amyloid‐lowering effects on specific soluble tau biomarkers and use the stage of the disease to assess tau‐monotherapy and sequential amyloid/tau therapies on disease‐related biomarkers. The DIAN‐TU platform serves as a model for international collaborative platform trials in genetic AD,^134^ producing groundbreaking results in secondary prevention.

Addressing rare tauopathies such as MAPT‐FTD necessitates international collaboration to build sufficiently large cohorts for effective drug trials. One example is the FTD Prevention Initiative, which has been instrumental in building trial‐ready cohorts and models of presymptomatic markers of the disease that together improve the design and feasibility of clinical trials.^135^ Basket trials might also be helpful in other tauopathies,^136^ where one drug is tested across multiple diseases with a shared element in pathology, increasing the efficiency of drug development by enhancing recruitment, sharing placebo groups, and enabling comparison across different tauopathies in the context of efficacy and side‐effect profiles.

Finally, it should be noted that engaging participants in the co‐design of trials is critical to enhance recruitment, ensure individual support, and maintain engagement and retention throughout the lengthy and demanding trial process. The challenges unique to different tauopathies must be addressed by tailoring trial designs to meet the specific demands of each condition.

In summary, refining the clinical trial design in tauopathies involves leveraging lessons from anti‐amyloid trials, utilizing evolving biomarkers for target engagement and disease modification, and considering trial platforms tailored to each tauopathy. Global collaboration coupled with participant engagement holds the keys to accelerating therapeutic advances in this devastating group of diseases, bringing us closer to meaningful clinical advancements.

## 8 TAUOPATHY‐DIRECTED THERAPEUTICS

Therapies to treat tau neurodegeneration have long been elusive in drug development. Not only are most neurodegenerative diseases multifactorial, making it challenging to select a single target, but the blood–brain barrier (BBB) also presents a major challenge for drug delivery. Despite these challenges, recent years have witnessed a surge of interest in lowering total tau as a therapeutic avenue.

Tau knockdown in adult animals has now been assessed genetically,^137^ with AAV tau zinc‐finger transcription factors,^138^ and with antisense oligonucleotides (ASOs).^139^, ^140^ Together, preclinical tau knockout and tau knockdown data demonstrate that lowering endogenous tau in adult animals is safe over long durations,^138^ lowering endogenous tau protects against neuronal hyperexcitability^139^ and dystrophic neurites,^139^ and lowering human tau protects against neuronal death^137^ and reverses preexisting tau pathology.^140^ Translation of these findings into clinical trials, such as the development of MAPT ASO (BIIB080) for AD has yielded encouraging results, including a favorable safety profile in humans, significant CSF tau protein reduction (∼60%), and, remarkably, a reversal of pathological tau PET signal.^128^

However, challenges remain, particularly regarding the inability of ASOs to cross the BBB, requiring repeat intrathecal administrations. To address this limitation, new approaches are in preclinical development, such as the conjugation of ASO to a human transferrin receptor (TfR) binding biologic to enable the transport of ASO across the BBB with intravenous (IV) dosing.^141^ Taking advantage of high TfR levels at the BBB, this oligonucleotide transport vehicle (OTV) platform uniformly distributes functional ASO throughout the CNS in mice and non‐human primates.^142^

Alternative therapeutic strategies, such as tau intrabodies, monoclonal antibodies such as bepranemab, senolytics, and O‐GlcNAcylation inhibition, are actively being explored, with preclinical and clinical studies showing encouraging safety profiles and efficacy in targeting tau pathology and mitigating neurodegeneration.^143^ These advances underscore a growing momentum in pursuing effective treatments for neurodegenerative diseases, as summarized below.

### 8.1 Tau intrabodies

Significant efforts have focused on developing tau‐lowering strategies aimed at targeting extracellular tau to disrupt neuron‐to‐neuron spread. However, these therapeutics have yet to prove effective in altering disease progression. A promising alternative approach involves using intrabodies – intracellular antibodies – to target and degrade tau inside cells.^144^, ^145^ In line with this, researchers at the Neural Stem Cell Institute in New York hypothesized that reducing monomeric tau through normal protein clearance pathways would mitigate abnormal tau accumulation, thereby alleviating cell toxicity and neurodegeneration.^146^ Their approach utilizes bifunctional intrabodies to bind tau, preventing aggregation and targeting soluble monomers to the proteasome for degradation. Fusion with the ornithine decarboxylase (ODC) proline, glutamic acid, serine, and threonine degron (PEST sequence) enables ubiquitin‐independent proteolysis of intrabody‐bound tau by directly interacting with the proteasome. By leveraging this direct molecular interaction with the proteasome, the degradation process is controlled through programmable target antigen proteolysis technology (PTAP). Seventeen human anti‐tau‐PEST intrabodies were engineered, selecting candidates based on their efficacy in reducing enhanced Green Fluorescent Protein (eGFP)‐labeled 0N4R‐tau in an immortalized rat neuronal progenitor cell line (ST14A), with three achieving reductions in eGFP‐tau of approximately 78%, 70%, and 74% after 72 h of treatment. When the proteasome was inhibited using epoxomicin or MG132, tau levels significantly increased compared to vehicle‐treated controls across all groups. Treatment of induced Pluripotent Stem Cells (iPSC)‐derived 2‐month‐old cortical organoids over 21 days showed significant reductions in endogenous tau, with anti‐tau intrabodies of V‐PEST, N‐PEST, and F‐PEST exhibiting reductions of 55.3%, 57.9%, and 38.82%, respectively. These findings underscore the potent efficacy of anti‐tau‐PEST intrabodies in reducing tau levels in cell lines and 3D organoid models.^146^ Furthermore, a partial reduction of tau by PTAP is as effective as a high reduction of tau to counteract cell death in V337 M cultures.^146^ Currently, the in vivo efficacy of anti‐tau PTAP intrabodies is being evaluated using the hTau.P301S transgenic mouse model, building on previous studies with bifunctional anti‐synuclein‐PEST intrabodies.^147^, ^148^

### 8.2 Bepranemab: overview of Phase I/IB clinical study program

Bepranemab is a recombinant, humanized, full‐length immunoglobulin G4 monoclonal antibody (mAb) that targets a central epitope (amino acids 235‐250) proximal to the MTBR within tau.^149^, ^150^ The MTBR is thought to be responsible for tau aggregation and is less likely to be lost due to post‐translational cleavage than other epitopes, including the N‐ or C‐terminal adjacent epitopes^151^ that were the target of previously failed tau mAbs. In preclinical mouse models of the disease, bepranemab prevented the induction of tau pathology and blocked tau spread throughout the brain.^149^, ^152^

Three Phase I double‐blind, randomized, placebo‐controlled studies assessing the safety and tolerability of bepranemab have been completed: (1) UP0047 (ClinicalTrials.gov number NCT03464227) was a study of 52 healthy male volunteers aged 18 to 75 years, who were recruited at a single center in Germany. Bepranemab and placebo were administered in single ascending doses via IV infusion to assess safety, pharmacokinetics, and pharmacodynamics; (2) UP0065 (ClinicalTrials.gov number NCT03605082) was a study of 24 healthy volunteers of Japanese descent, aged 20 to 75 years old, who were recruited at a single center in the United Kingdom. Bepranemab and placebo were administered as single repeat doses via IV infusion to assess safety, tolerability, and pharmacokinetics; and (3) PSP003 (ClinicalTrials.gov number NCT04185415) was a multicentre Phase Ib study of 25 participants with PSP. Participants were >40 years old, met the criteria for probable PSP‐RS, and could walk at least five steps with minimal or no assistance. Bepranemab and placebo were administered as single repeat doses via IV infusion to assess safety and tolerability. Overall, bepranemab was well tolerated with an acceptable safety profile, thus supporting the progression of the clinical development of bepranemab in people with tauopathies.^153^ A Phase II study of bepranemab in people with early‐stage AD (ClinicalTrials.gov number NCT04867616) is currently under way.

### 8.3 Senolytics

Cellular senescence refers to the end stage of a complex stress response that allows damaged cells to survive in a toxic state.^154^ Therapeutically clearing senescent cells with “senolytics” has emerged as a promising approach to prevent, delay, and/or treat age‐associated diseases, including tauopathies.^155^ Intraneuronal tau accumulation is associated with senescent cells in *post mortem* PSP and AD brains.^156^, ^157^ Mechanistic studies in mice show that aberrant tau is causal in promoting cellular senescence; removing these senescent cells with senolytics leads to improved clinically relevant outcomes.^156^ These promising preclinical data led to the first Phase I trial testing these senolytics (dasatinib plus quercetin, known as D+Q) in the context of AD. The treatment was safe and well tolerated, and levels of dasatinib were detected in the CSF.^158^ Disease modification could not be inferred with the small sample (*n* = 5); however, biomarkers of disease progression were stable across the 3‐month study with some suggestions of changes in pathogenesis.

Additional ongoing AD trials are testing D+Q. These include two Phase I trials (ClinicalTrials.gov numbers NCT04785300 and NCT05422885) and a multi‐site Phase II randomized, placebo‐controlled trial (ClinicalTrials.gov number NCT04685590). The Phase II trial, SToMP‐AD,^159^ is currently half enrolled with an anticipated completion of late 2025. Beyond D+Q, many other senolytics are in the drug discovery pipeline for treating neurodegenerative diseases, highlighting the excitement for advancing this rapidly evolving field.

### 8.4 O‐GlcNAcylation inhibition

O‐GlcNAcylation, a post‐translational modification (PTM) of proteins, has recently emerged as a potential regulator of diverse cellular functions. This modification involves the glycosylation of serine and threonine residues with O‐GlcNAc monosaccharide and is exclusively regulated by two highly conserved enzymes: O‐GlcNAc transferase (OGT), which adds GlcNAc onto target proteins, and O‐GlcNAcase (OGA), which removes GlcNAc.^160^ Interestingly, O‐GlcNAcylation and phosphorylation can occur on the same serine and threonine residues, suggesting a complex antagonistic crosstalk between these PTMs. This implies that O‐GlcNAcylation may act as a protective layer against the phosphorylation of various proteins like tau.^161^ Additionally, O‐GlcNAcylation of tau proteins at Serine 400 has been shown to directly inhibit aggregation.^162^

Under basal conditions, there is an equilibrium between PTMs, but when hyperphosphorylation is promoted, tau tends to detach from microtubules and self‐aggregate into NFTs (a toxic insoluble form of tau).^163^ Such aggregates are a common feature of several tauopathies, like PSP. A new promising therapeutic approach for PSP is OGA inhibition: Pharmacological blockade of OGA would result in an increase of soluble glycosylated tau, inhibiting the formation of pathological tangles.

Pharmacological OGA inhibition has already demonstrated therapeutic potential in different preclinical models, providing a strong rationale for the development of OGA inhibitors as disease‐modifying agents in tauopathies.^162^ FNP‐223, a novel oral selective OGA inhibitor, has shown safety and efficacy in reducing tau aggregates in preclinical models.^164^ Moreover, it has demonstrated favorable safety, pharmacokinetics, and brain penetration in clinical Phase I studies, being well tolerated with no dose‐limiting toxicities or serious adverse events.^106^ Based on these results, researchers are developing an RDBPC Phase II study to assess the clinical efficacy, safety, and pharmacokinetics of FNP‐223 to slow the progression of PSP (PROSPER study, ClinicalTrials.gov number NCT06355531).

## 9 CONCLUSION

The Tau2024 Global Conference showcased the remarkable progress made in understanding and addressing tau‐associated brain pathologies, already leading to better diagnostics and therapeutic strategies. With registrants spanning 58 countries across Africa, Asia, Europe, North America, Latin America, and Oceania, the conference served as a global meeting. Studies presented from around the world underscore the importance of addressing socioeconomic and genetic disparities in understanding tau disorders among underserved populations and the necessity for international collaboration. Recent breakthroughs in biomarker developments, such as blood‐based biomarkers for AD that offer enhanced specificity and longitudinal monitoring capabilities, were presented. Despite these advancements, challenges remain, such as assay specificity and physiological confounders, driving innovation toward more accessible diagnostic approaches. Many targetable mechanisms have emerged in the cascade of pathogenesis for tauopathies. These include interactive processes underlying DNA structure, RNA translation, post‐transcriptional modification by OGA, and phosphorylation through to trans‐synaptic transmission and astrocyte‐mediated synapse engulfment, neuroinflammation, and senescence. These novel insights into disease pathogenesis are leading to new therapeutic targets and diversifying the therapeutic pipeline. Improved clinical trial designs, biomarker development, and therapeutic optimism are energizing the pipeline of discoveries needed to deliver approved treatments for tauopathies. Through international, collaborative efforts, innovative research, and a commitment to inclusivity, researchers worldwide have demonstrated transformative breakthroughs toward diagnosing, treating, and ultimately preventing tau‐related diseases.

## CONFLICT OF INTEREST STATEMENT

M.C. Carrillo, I. Camargo Fontana, S. Mahinrad, and C. Sexton are full‐time employees of the Alzheimer's Association. N.J. Ashton, in the past 36 months, reports receiving consulting fees from Quanterix; payment or honoraria for lectures, presentations, speaker bureaus, manuscript writing, or educational events from Alamar Biosciences, Biogen, Eli‐Lilly, Quanterix; patents (Application No.: PCT/US2024/037834 [WSGR Docket No. 58484‐709.601]), and served as Advisory Board for Biogen, TargetALS, and TauRx. L. Jacobs, in the past 36 months, reports receiving travel and lodging support for being the united keynote speaker by Alzheimer's Association and has a leadership or fiduciary role in Cure MAPT FTD. M. B. Maina, in the past 36 months, reports receiving consulting fees from Wellcome Trust; serving as a Member of Council, International Society to Advance Alzheimer's Research and Treatment; serving as Ambassador, ALBA Network (https://www.alba.network/); Governing of Society of Neuroscientists of Africa (SONA) and Yobe State Government–Special Adviser to The Governor. O. Hansson, in the past 36 months, reports receiving consulting fees from AC Immune, BioArctic, Biogen, Bristol Meyer Squibb, C2N Diagnostics, Eisai, Eli Lilly, Fujirebio, Merck, Novartis, Novo Nordisk, Roche, Sanofi, and Siemens. M. Malpetti, in the past 36 months, reports receiving consulting fees from Astex Pharmaceuticals, Alzheimer's Association Travel Grant, Guarantors of Brain Travel Grant, ARUK East Network Travel Grant; and served as lead of the Inflammation Special DEMON Group, and the PET GENFI working group. R. Yadav, in the past 36 months, reports receiving royalties or licenses from Jaypee Publishers, New Delhi, received payment or honoraria for lectures, presentations, speaker bureaus, manuscript writing, or educational events from International PD and Movement Disorders Society, and served as Secretary of Movement Disorders Society of India. B. Frost, in the past 36 months, reports receiving consulting fees from MD Anderson Belfer Neurodegeneration Consortium, paid travel by Rainwater Foundation for attending Tau2024, Washington, D.C., paid travel by MD Anderson for attending MD Anderson Belfer Neurodegeneration Consortium Science Day, Houston, TX, and paid travel by NIH for attending CMND Study Section, Washington, D.C.; served as Co‐organizer of Tau2024 Conference, Scientific Advisory Board of CurePSP and Associate Editor of Progress in Neurobiology. L. Buee, in the past 36 months, reports receiving support from Rainwater Charitable Foundation for attending the Global Tau2024 Conference. D. Butler, in the past 36 months, reports support from the Rainwater Charitable Foundation and the Tau Consortium for attending meetings and/or travel and patents (Regenerative Research Foundation (2018). BI‐FUNCTIONAL ANTI‐TAU POLYPEPTIDES AND USE THEREOF. 27562‐0024WO1, Regenerative Research Foundation (2021) COMPOSITIONS AND METHODS FOR CONTROLLED PROTEIN DEGRADATION IN NEURODEGENERATIVE DISEASE (Pending)). C. D. Clelland, in the past 36 months, reports receiving support for attending Tau2024 and TCIM 2024. C. Ewen reports being an employee of UCB Ltd; receiving travel/hotel support for Tau2024 from the Rainwater Foundation; and stock or stock options in UCB Ltd. R. M. Edelmayer is a full‐time employee of the Alzheimer's Association, and the Alzheimer's Association provided support for the Tau2024 conference and writing of the manuscript, and Alzheimer's Association paid for her travel to Tau2024. K. Horie may receive income based on technology (METHODS TO DETECT MTBR TAU ISOFORMS AND USE THEREOF) (PCT/US2020/046224) licensed by Washington University to C2N Diagnostics. K. Horie may receive income based on technology (ANTI‐TAU MTBR ANTIBODIES AND METHODS TO DETECT ENDOGENOUSLY CLEAVED FRAGMENTS OF TAU AND USES THEREOF) (PCT/US2023/072738) licensed by Washington University to C2N Diagnostics. In addition, K. Horie reports the following patents: ANTI‐TAU MTBR ANTIBODIES AND METHODS TO DETECT ENDOGENOUSLY CLEAVED FRAGMENTS OF TAU AND USES THEREOF (PCT/US2023/072738), METHODS TO DETECT MTBR TAU ISOFORMS AND USE THEREOF (PCT/US2020/046224), and reports being an Eisai‐sponsored voluntary research associate professor at Washington University and has received a salary from Eisai. J. B. Rowe, in the past 36 months, reports royalties or licenses from Oxford University Press; receiving consulting fees from Asceneuron, Astex, Astronautx, ClinicalInk, CumulusNeuro, Cerevance Curasen, Eisai, ICG, Invicro, and Prevail; serving on the advisory board of Asceneuron and Dementia Mission; and being trustee of Guarantors of Brain, Darwin College, PSP Association. T. L. S. Jones, in the past 36 months, reports receiving consulting fees from AbbVie and Jay Therapeutics; receiving payment or honoraria for lectures, presentations, speaker bureaus, manuscript writing, or educational events from Sanofi; support for attending meetings and/or travel from Sanofi; and serving as president and trustee of British Neuroscience Association, Guarantors of Brain, trustee and editor of Brain Communications, Charity Scientific Advisor of Race Against Dementia, Scientific Advisory Board of Scottish Brain Sciences, and Scientific Advisory Board Cognition Therapeutics. C. J. Mummery, in the past 36 months, received consulting fees from Lilly as expert advisor in development of clinical program of siRNA J4T‐MCL‐0LAA; received honoraria for sponsored symposia: (a) Scientific symposium on novel DMTs in dementia; (b) educational symposium on DMTs from Lilly; received honoraria for sponsored symposium at ABN on implementation of leqembi in UK from Eisai; received paid registration fee and travel for UK National neurology conference (ABN) from Esiai, and paid registration and travel for AAIC as a Scientific Program Committee from Alzheimer's Association; and reports the following: Lilly – member of advisory board on donanemab Trailblazer; Novartis – member of advisory board on AD drug program steering committee; Roche/Genentech – member of advisory board for trontinemab, Eisai – member of advisory board on UK AUR for Leqembi, Chair data safety monitoring board Immunobrain, Biogen – advisor on program steering committee EMBARK/ENVISION aducanumab, Biogen – advisor on program steering committee and PI for phase II CELIA BIIB080, and Eisai – chair of AUR development UK committee for Leqembi. C. Sastré reports being Ferrer Employee. M. C. Galas, in the past 36 months, reports being an invited guest at the Tau2024 Global Conference. K. Diaz reports serving as Executive Director and CSO of CurePSP, Inc. K. Wildsmith, in the past 36 months, reports receiving support for attending meetings and/or travel from Eisai, Inc. and stock or stock options at Eisa, Inc. B. L. Tee, in the past 36 months, reports support for attending meetings and/or travel from NIA (R21AG068757, R01AG080469) and Alzheimer's Association (AACSFD‐22‐97214). F. M. Elahi, in the past 36 months, reports receiving consulting fees from Back Bay Life Science Advisors, Woolsey Pharmaceuticals, and Therini; payment or honoraria for lectures, presentations, speaker bureaus, manuscript writing, or educational events from academia only; support for attending meetings and/or travel from academia only; and serving on the cureCADASIL scientific advisory board, Eisai Alzheimer's disease advisory board, scientific advisory board member, Albert White Matter Research Institute, and scientific advisory board member and consultant, Cordance Medical. E. McDade, in the past 36 months, reports receiving consulting fees from AstraZeneca, Roche, Sanofi, and Merck; receiving payment or honoraria for lectures, presentations, speaker bureaus, manuscript writing, or educational events from Alzheimer Association, Projects in Knowledge (Kaplan)‐ CME, and Neurology Live–CME; receiving support for attending meetings and/or travel from Alzheimer's Association and Alzheimer's Foundation; patents planned, issued, or pending (T‐018562 – methods of treating based on site‐specific tau phosphorylation); participation on a data safety monitoring board or advisory board of Alector and Alnylam Pahrmaceuticals; having a leadership or fiduciary role paid or unpaid in Alzamend; and receiving equipment, materials, drugs, medical writing, gifts, or other services from Avid Radiopharmaceuticals, Cerveau, and LMI. G. Höglinger, in the past 36 months, reports receiving consulting fees from Abbvie, Alzprotect, Amylyx, Aprinoia, Asceneuron, Bayer, Bial, Biogen, Biohaven, Epidarex, Ferrer, Kyowa Kirin, Lundbeck, Novartis, Retrotope, Roche, Sanofi, Servier, Takeda, Teva, UCB; receiving payment or honoraria for lectures, presentations, speaker bureaus, manuscript writing, or educational events from Abbvie, Bayer, Bial, Biogen, Bristol Myers Squibb, Esteve, Kyowa Kirin, Pfizer, Roche, Teva, UCB, Zambon; receiving support for attending meetings and/or travel from Alzheimer's Association, CurePSP, Deutsche Gesellschaft für Neurologie, Deutsche Parkinson Gesellschaft, European Academy of Neurology and Movement Disorders Society; having patents planned, issued, or pending (Höglinger GU, Höllerhage M, Rösler T. Treatment of Synucleinopathies, United States Patent No.: US 10,918,628 B2, date of patent: February 16, 2021, and Höglinger GU, Höllerhage M, Rösler T. Treatment of Synucleinopathies. European Patent No.: EP 17 787 904.6‐1109 / 3 525 788.); participation on a Data Safety Monitoring Board or Advisory Board of Kainos Medicine; and having the following leadership or fiduciary role in other board, society, committee, or advocacy group, paid or unpaid: scientific advisory board, CurePSP, scientific advisory board, Parkinson Stiftung, scientific advisory board, Thiemann Stiftung, and clinical advisory board, DZNE. M. Brendel, in the past 36 months, reports receiving consulting fees from MICA and GE Healthcare; receiving payment or honoraria for lectures, presentations, speaker bureaus, manuscript writing, or educational events from GE Healthcare, Miltnei, and Life Molecular Imaging; participation in a data safety monitoring board or advisory board of MIAC, and GE Healthcare; and having the following leadership or fiduciary role in other board, society, committee, or advocacy group, paid or unpaid: EANM Neuroimaging, and SNMMI BIC Board of Directors. S. L. DeVos, in the past 36 months, reports the following patens planned, issued, or pending: DeVos SL, Miller TM, Rigo F, Bennett CF. Methods for modulating tau expression for reducing seizure and modifying a neurodegenerative syndrome. US20200032257A1. Published 01/2020. Patent Granted 10/2023, DeVos SL, Miller TM, Rigo F, Bennett CF. Methods for modulating tau expression for reducing seizure and modifying a neurodegenerative syndrome. US10273474B2. Published 10/2015. Patent Granted 04/2019, DeVos SL, Barker S, Dennis MS, Estrada A, Kariolis M, Mahon C, Nilewski L, Park J, Shan L, Thayer MB, Tong R, Tran H, Wells R, Zuchero J. Oligonucleotide conjugates targeted to the TfR. WO2023279099A1. Published 01/2023. Patent Pending, DeVos SL, Ledeboer A, Zeitler B, Zhang S, Wegmann S, Hyman B. Tau modulators and methods and compositions for delivery thereof. US11504389B2. Published 06/2018. Patent Granted 11/2022, and DeVos SL, Ledeboer A, Zeitler B, Zhang S, Wegmann S, Hyman B. Tau modulators and methods and compositions for delivery thereof. US20230270774A1. Published 08/2023. Patent Pending. In addition, S. DeVos, in the past 36 months, reports having stock or stock options in Denali Therapeutics, Inc. J. D. Rohrer, in the past 36 months, reports receiving consulting fees from Alector, Prevail, Aviado Bio, Denali, Arkuda Therapeutics, and Takeda. J. F. Abisambra, in the past 36 months, reports receiving support for attending AAIC 2022. M. Orr, in the past 36 months, has received research support related to the present manuscript from Hevolution/AFAR, Cure Alzheimer's Fund, NIH grant 5R01AG068293‐05, the Alzheimer's Drug Discovery Foundation, and the Rainwater Charitable Foundation (these awards were granted to WFUHS, with Dr. M. E. Orr serving as the Principal Investigator). In addition, M. E. Orr received consulting fees from the Foundation for a Better World, received an honorarium for a keynote speech at a retreat at Case Western Reserve University, and an honorarium for a lecture from Washington University and Northwestern University. She received support for attending the following meetings and/or travel: AGE 51st Annual Meeting, AAIC 2023, 46th Annual Meeting of the Japanese Neuroscience Society, FBI Translations Seminar Series, CTAD 2023, ISCA 2023, GSA 2023, SfN 2023, Multidisciplinary Research in Gerontology Colloquium Series at USC, Progress in Neuroscience Seminar Series (PINS), AGBT 2024, ADPD 2024, ICM Seminar, Tau 2024, Palm Beach Science Series 2024, Japan Spatial Summit, ISMND 2024, Spatial Biology East Coast Summit, FrA2RE Meet and Greet Event, IMPACT‐AD Alumni Scholar Meeting, IRCND 2024, ADDF Pre‐AAIC Gathering, 2024 Neurobiology of Brain Disorders Gordon Research Conference, Case Western Annual Neurodegeneration Retreat and Goodes Prize Anniversary. She served as an unpaid advisor to Nanostring Technology and received free lab services from Nanostring Technology, Canopy Bio, and Averill Foundation. A. Rommel is an employee at Rainwater Charitable Foundation, which was a co‐funder of the Tau2024 conference. C. Sastre is a Ferrer employee. The remaining authors report no disclosures. Author disclosures are available in the Supporting Information.

## Supporting information



Supporting Information
